# Bioturbation by endogeic earthworms facilitates entomopathogenic nematode movement toward herbivore-damaged maize roots

**DOI:** 10.1038/s41598-020-78307-0

**Published:** 2020-12-04

**Authors:** Sandrine Fattore, Zhenggao Xiao, Adrienne L. Godschalx, Gregory Röder, Ted C. J. Turlings, Renée-Claire Le Bayon, Sergio Rasmann

**Affiliations:** 1 Laboratory of Functional Ecology, University of Neuchâtel, Rue Emile-Argand 11, 2000 Neuchâtel, Switzerland; 2 Institute of Environmental Processes and Pollution Control, School of Environmental and Civil Engineering, Jiangnan University, Wuxi, 214122 China; 3 Fundamental and Applied Research in Chemical Ecology, Institute of Biology, University of Neuchâtel, Rue Emile Argand 11, 2000 Neuchâtel, Switzerland

**Keywords:** Agroecology, Community ecology

## Abstract

Entomopathogenic nematodes (EPNs) have been extensively studied as potential biological control agents against root-feeding crop pests. Maize roots under rootworm attack have been shown to release volatile organic compounds, such as (*E*)-β-caryophyllene (Eβc) that guide EPNs toward the damaging larvae. As yet, it is unknown how belowground ecosystems engineers, such as earthworms, affect the biological control capacity of EPNs by altering the root Eβc-mediated tritrophic interactions. We here asked whether and how, the presence of endogeic earthworms affects the ability of EPNs to find root-feeding larvae of the beetle *Diabrotica balteata*. First, we performed a field mesocosm experiment with two diverse cropping systems, and revealed that the presence of earthworms increased the EPN infection potential of larvae near maize roots. Subsequently, using climate-controlled, olfactometer-based bioassays, we confirmed that EPNs response to Eβc alone (released from dispensers) was two-fold higher in earthworm-worked soil than in earthworm-free soil. Together our results indicate that endogeic earthworms, through burrowing and casting activities, not only change soil properties in a way that improves soil fertility but may also enhance the biocontrol potential of EPNs against root feeding pests. For an ecologically-sound pest reduction in crop fields, we advocate agricultural practices that favour earthworm community structure and diversity.

## Introduction

Agricultural intensification can produce high crop yields, but often comes at the cost of significant land and biodiversity degradation, ultimately causing major concerns for long-term food production^1^. Furthermore, monocultures are prone to infestation as pathogens and pests can rapidly spread among host plants that are genetically homogeneous and spatially compact. To counterbalance the negative effects of intense crop farming, focus has turned to more sustainable practices, including organic agriculture (i.e. eliminating the use of synthetic inputs, and promoting soil biodiversity and biological activity), and intercropping cultivation (i.e. growing two or more mutually beneficial crops), which allow minimum disturbance to agroecosystems and maximum preservation of soil biodiversity, functions and services^2^. Sustainable farming also goes hand in hand with other complementary approaches such as integrated pest management (IPM), which focuses on the use of biological control agents to control and reduce pest populations.

Many of the most important agricultural pests are exotic and jeopardise crops because they have been accidently introduced into new environments without their natural enemies. For instance, *Diabrotica virgifera virgifera*, or western corn rootworm (WCR), is a major maize pest in the USA, as well as in Central Europe since its invasion in the early 1990’s. Many strategies have been proposed to reduce WCR outbreaks, including crop rotation, foliar and soil insecticides, breeding for higher tolerance, genetic engineering, as well as the use of natural enemies for the biological control of the root-feeding larvae^3^. Among the most promising biocontrol agents of WCR are the soil-dwelling entomopathogenic nematodes (EPNs)^4^. EPNs can actively ‘hunt’ for hosts, they can kill the hosts in a few days, and they can be easily mass-produced. Previously, it was shown that when attacked by rootworms, the roots of several maize varieties release (*E*)-β-caryophyllene (hereafter referred to as Eβc), a sesquiterpene volatile organic compound that is highly attractive to EPNs such as *Heterorhabditis megidis*^5^. Therefore, planting a maize variety that emits Eβc while also managing crops in a way that promotes healthy soil ecosystems that support biocontrol interactions should reduce rootworm damage in maize fields, and thus reduce the need for synthetic pesticides.

Crafting healthy soil ecosystems requires further understanding of complex plant-soil interactions. Indeed, contrary to root-feeding pests, soil-dwelling inhabitants can also generate positive relationships with plants. For instance, overwhelming evidence shows that earthworms positively affect plant performance^6^. Via their feeding, burrowing and casting activities, earthworms can improve soil aeration and soil water retention capacity, promote nutrient turnover and stimulate beneficial microbial biomass and activity, thus promoting plant growth^7^. Recent research also suggests that earthworms can influence the outcome of plant–herbivore interactions^8^. The effects of earthworms on plant–herbivore interactions can be summarized as follows: first, burrowing earthworms come into direct contact with the crop root system, which can cause external force stimulation on roots and even mechanical root damage, which directly activate plant defence systems^9^. Second, earthworms can indirectly influence plant–herbivore interaction by changing the physico-chemical properties of the soil^9^. Because soil nutrient content and fertility can influence plant defensive strategies^10^, earthworms can thus indirectly modify plant defence responses to herbivore attack. Third, earthworms can indirectly affect root feeders by increasing the dispersal and biocontrol capacity of entomopathogenic nematodes (e.g. *Steinernema* sp.)^11,12^. In addition, earthworms’ epidermis secretes mucus, which is mainly composed of water, polysaccharides and amino-acids. Previous studies have shown that earthworm mucus could alter soil bacterial diversity and activities, and interfere with the behaviour and physiology of soil-dwelling bacterial-feeding nematodes (*Mesorhabditis* sp. and *Protorhabditis* sp.)^13^. Thus, earthworms’ secretions might be similar to root exudates, acting as important drivers of belowground tritrophic interactions.

**Figure 1:**
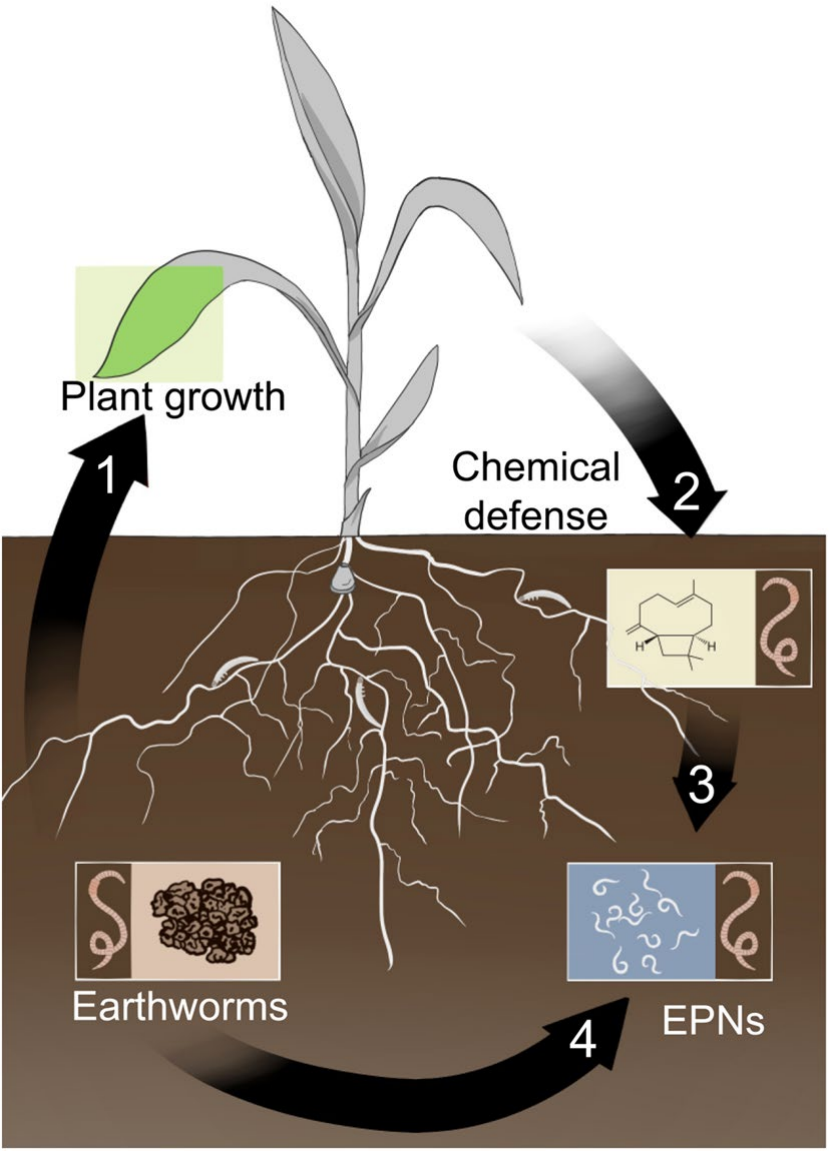
Graphical abstract. A semi-field experiment and several olfactometer-based experiments were conducted to address the effect, and the mechanisms, respectively, of earthworms on belowground tritrophic interactions between maize plants, rootworm larvae and entomopathogenic nematodes (EPNs). Particularly, we hypothesized that the borrowing and casting activity of earthworms would promote soil fertility, and thus plant growth (1), and also enhance chemical defence production (2), which in turn would stimulate EPNs recruitment (3). Finally, we hypothesized that soil labouring by earthworms would generate a more favourable soil environment for EPN movement and host seeking behaviour (4).

Endogeic earthworms are particularly interesting in the agroecological context, as they inhabit the first 20–30 cm of soil, where much of the root system of plants lies along with rhizospheric microbial activity. Endogeic species are generally active, as they need to ingest large amounts of soil to meet their nutritional needs. They are known to form a branched burrow system that they do not necessarily reuse, thus creating a favourable environment for root penetration, aeration and nutrient uptake^14^. The aim of this study was to measure the effect of endogeic earthworms on EPN recruitment to roots under herbivore attack, and to address potential mechanisms driving EPN-earthworm interactions. Specifically, we asked whether endogeic earthworms affect the efficacy of EPNs as biocontrol agents, and if so, if this effect is more pronounced in a monoculture compared to a polyculture. To address these questions, we first conducted a semi-field mesocosm experiment and measured the infectivity of the EPN *H. megidis* in the presence or absence of the endogeic earthworm *Allolobophora icterica* and in mono- or diverse cropping systems. We predicted that crop diversity and earthworm bioturbation activity would interactively enhance plant growth via nutrient-rich soil. Gouinguené and Turlings^15^ revealed that maize plants watered with an N-rich nutrient solution released more defence-related volatiles than unfertilized plants. Similar effects could be expected from earthworms’ soil enrichment alongside bean plant nitrogen-inputs, potentially having a double positive impact on maize bioprotection. We therefore predicted maize plants in earthworm-worked polycultural systems to attract more EPNs in response to herbivore damage signals. We further wondered if earthworm-EPN interactions are directly mediated by changes in the physicochemical properties of the soil, or through direct or indirect changes in plant secondary metabolism. To address this question, we conducted three independent olfactometer-based laboratory experiments, in order to dissect the mechanisms driving earthworm’s effects on tritrophic interactions (Fig. 1).

## Results

### Mesocosm experiment

**Figure 2:**
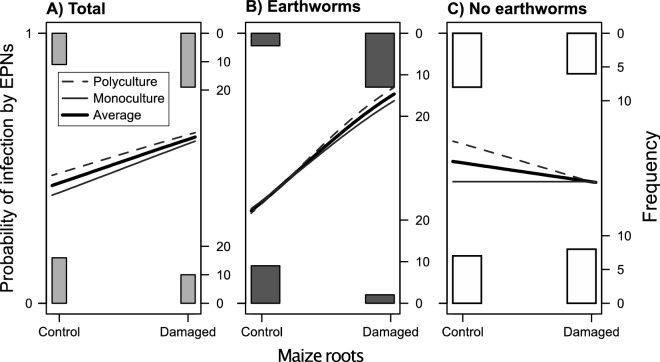
Probability of infection by EPNs. Solid black lines show the probability of a *Galleria mellonella* larva being infected by *Heterorhabditis megidis* EPNs when placed close to a healthy root system (Control), or a mechanically-damaged root system of maize (*Zea mays* var. Delprim) plants and induced with JA. Dashed and solid grey lines show the same effect, but the dataset is divided between the polyculture (maize grown with squash and bean plants), or the monoculture (only maize) treatments, respectively. Panel (**A**) shows the overall effect, while panels (**B**) and (**C**) show the probabilities of EPN infection in mesocosms with (dark grey boxes), or without (open boxes) *Allolobophora icterica* earthworms, respectively*.*

Overall, we found that the induction treatment with jasmonic acid (JA) on roots of maize plants significantly increased the probability of a *G. mellonella* sentinel larva of being infected (Fig. 2A; Table 1). This effect was independent of the type of culture (monoculture *versus* polyculture) in the mesocosms, but was influenced by the presence of earthworms. Particularly, when *A. icterica* earthworms were present in the mesocosm, the probability of *G. mellonella* larvae getting infected when placed close to an induced maize root was about 75% higher (Fig. 2B), than when the mesocosms were devoid of earthworms (Fig. 2C). We found that neither the type of culture, nor the earthworm presence changed soil cation exchange capacity (CEC) values (Supplementary Fig. S5A; culture type effect: F_1,34_ = 1.11, p = 0.30; earthworm effect: F_1,34_ = 1.84, p = 1.18, and their interaction: F_1,34_ = 0.30, p = 0.58). Soil carbon to nitrogen ratio (CN) was enhanced by 3% in monocultures (Supplementary Fig. S5B; culture type effect: F_1,34_ = 9.99, p = 0.003), as well as with earthworms by 2% (earthworms effect: F_1,34_ = 5.05, p = 0.03), but the presence of earthworms in monoculture increased soil CN value by 4% compared to all other treatments. Finally, plants gained 72% more total vegetative biomass (Supplementary Fig. S5C, Supplementary Table S1), and 28% more reproductive (corn earcobs) biomass (Supplementary Fig. S5D, Supplementary Table S1) in monocultures compared to polycultures, and there was no significant effect of earthworms on both vegetative and reproductive plant traits (Supplementary Table S1).

**Table 1:** Results from GLM (Generalized Linear Model) with quasi-binomial distribution for testing (A) the interactive effect of three factors: JA induction in root of maize (*Zea mays* var. Delprim) plants, the presence or absence of *Allolobophora icterica* earthworms in the soil, and the monoculture (only maize) or polyculture (maize + squash + bean) on *Galleria mellonella* sentinel larval infection by *Heterorhabditis megidis* EPNs.

Model	Factor	Estimate	Std. Error	t value	Pr( >|t|)
(A) Overall	(Intercept)	− 0.619	0.574	− 1.078	0.285
	JA induction (JA)	1.718	0.854	2.011	**0.048**
	Earthworm (EW)	0.418	0.795	0.526	0.601
	Culture (C)	− 0.074	0.840	− 0.088	0.930
	JA × EW	− 1.718	1.156	− 1.486	0.142
	JA × C	0.362	1.254	0.288	0.774
	EW × C	0.680	1.149	0.592	0.556
	JA × EW × C	− 0.990	1.682	− 0.589	0.558
(B) Polyculture	(Intercept)	− 0.693	0.635	− 1.092	0.283
	JA	2.079	0.953	2.183	**0.036**
	EW	1.099	0.860	1.278	0.210
	JA × EW	− 2.708	1.267	− 2.137	**0.040**
(C) Monoculture	(Intercept)	− 0.619	0.554	− 1.118	0.271
	JA	1.718	0.824	2.086	**0.044**
	EW	0.418	0.767	0.546	0.589
	JA × EW	− 1.718	1.114	− 1.542	0.132

Tables (B) and (C) show the same analyses but broken down by polyculture or monoculture mesocosms, and testing the interactive effect of JA induction and earthworms on EPNs infectivity.

### Four-arm olfactometer bioassay

**Figure 3:**
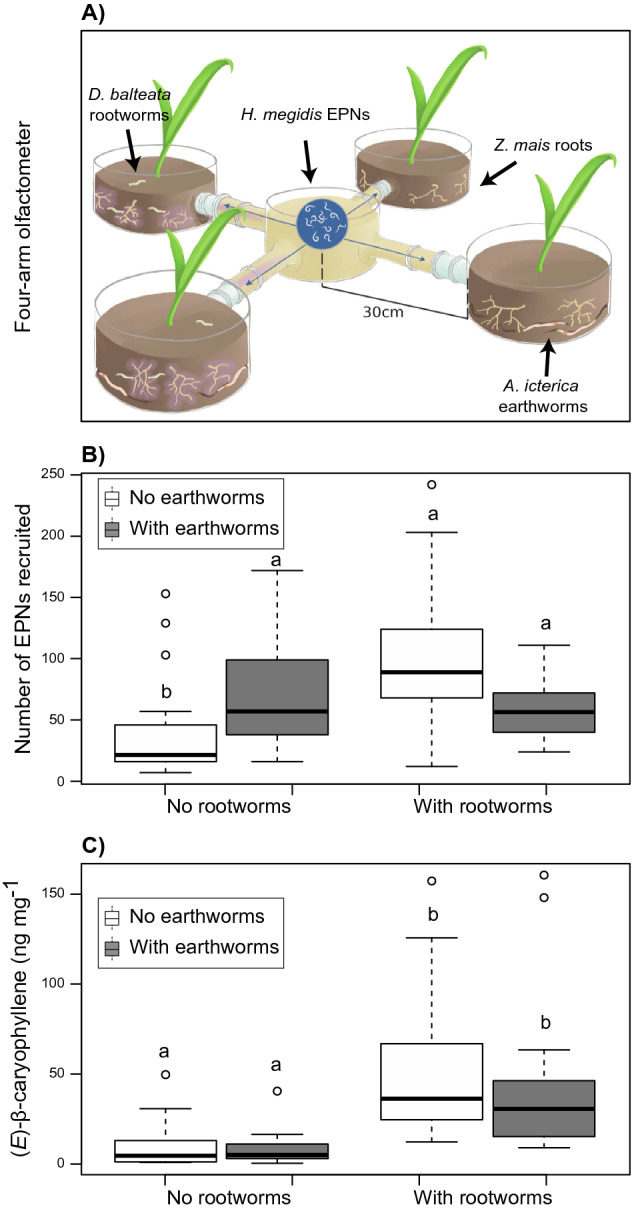
Four-arm olfactometer bioassay. (**A**) Schematic design of a four-arm olfactometer that fully-factorially manipulated the presence or the absence of *Allolobophora icterica* earthworms, and the presence or the absence of *Diabrotica balteata* larvae around seedlings of maize plants (*Zea mays* var. Delprim). The center of the arena, where *Heterorhabditis megidis* EPNs were released contained sand, whereas the side pots contained a mixture of sand and soil. (**B**) The number of *H. megidis* nematodes found in the arms of a four-arm olfactometer. (**C**) The amount of (*E*)-β-caryophyllene produced by the roots of the corn plants in the four treatments (n = 20). Letters above bars show significant differences among treatments obtained from contrasting marginal means after generalized linear model and linear model for panels (**B**) and (**C**), respectively.

We found a significant effect of the rootworm herbivory by earthworm treatments on the recruitment of EPNs in the four-arm olfactometer bioassay (Fig. 3A,B, earthworm effect: F_1,69_ = 0.14, p = 0.71; herbivore effect, F_1,68_ = 9.00, p < 0.001, and earthworm by herbivore interaction, F_1,65_ = 18.46, p < 0.001—experiment effect: F_2,66_ = 21.41, p < 0.001). Specifically, we found that in the absence of earthworms, insect-damaged roots of maize plants attracted 2.55 times more EPNs than healthy roots. Whereas in the presence of earthworms we found no difference in the attraction capacity of damaged and healthy plants, meaning that earthworms decreased EPN attraction by 40% when root herbivores were also present, but on the other hand, earthworms alone increased EPN attraction by 80% around undamaged roots (Fig. 3B). In the same experiment, we found that Eβc production was affected by rootworm herbivory but not by the presence of earthworms (Fig. 3C, root herbivory effect: F_1,64_ = 82.72, p < 0.001; earthworm effect: F_1,64_ = 0.15, p = 0.70; herbivory by rootworm interaction: F_1,64_ = 1.66, p = 0.20—experiment effect: F_2,64_ = 6.49, p = 0.002, and root biomass effect: F_1,64_ = 1.46, p = 0.23). Specifically, rootworm herbivory increased Eβc production in the maize roots 5.2-fold compared to undamaged roots (from 9.1 ng/g FW in healthy roots to 47.9 ng/g FW in damaged roots). Earthworms, on the other hand, increased average plant height by 14%, and total biomass by 18% (earthworm effect on the composite axis of biomass accumulation (PCA1 as described in Supplementary Fig. S4); F_1,65_ = 19.58, p < 0.001), while we found no significant effect of root herbivory on biomass accumulation (root herbivore effect: F_1,65_ = 0.25, p = 0.62, and earthworm by root herbivore interaction: F_1,65_ = 1.82, p = 0.18).

### Mucus experiment

We found that 51% more EPNs moved into the arms near roots that were not watered with earthworm mucus (Supplementary Fig. S6A; F_1,16_ = 18.05, p = 0.001). On the contrary, mucus-watered roots damaged by *D. balteata* larvae produced 28% more Eβc than control roots without mucus (Supplementary Fig. S6B; F_1,16_ = 8.44, p = 0.01), but we found no effect of mucus addition on root biomass (Supplementary Fig. S6C; F_1,16_ = 0.17, p = 0.69).

### Bioturbation and synthetic Eβc olfactometer bioassay

**Figure 4:**
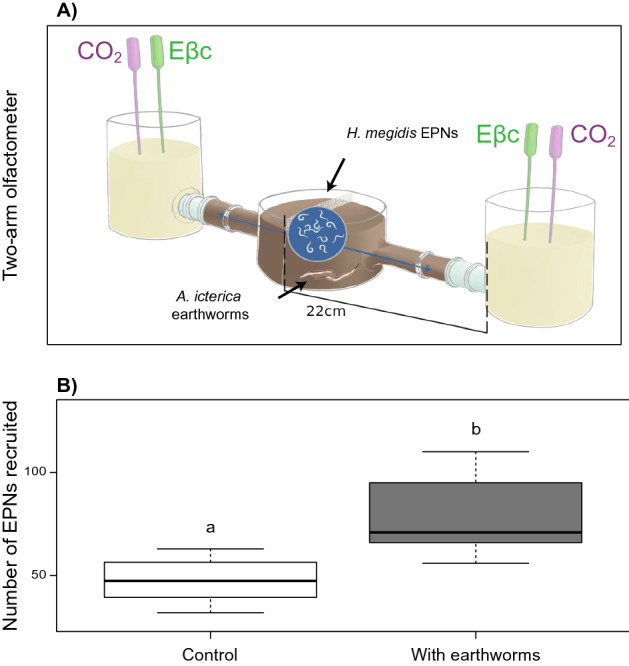
Earthworms-EPNs direct interaction bioassay. (**A**) Schematic design of a two-arm olfactometer that manipulated the presence or absence of *A. icterica* in the central soil where also EPNs are present. The side pots were inoculated with a mixture of synthetic CO2 and (*E*)-β-caryophyllene (Eβc) for stimulating EPN directional movement^22^. (**B**) Number of EPNs that were found in either sides of the two-arm olfactometer at the end of the bioassay (n = 10). Letters above bars show significant differences between treatments (linear model; p < 0.05).

The presence of earthworms (and earthworm-worked soil) doubled EPN attraction toward the Eβc + CO_2_ dispensers (from 48 EPNs in arms without earthworms to 98 EPNs in arms with earthworms in average, Fig. 4B; F_1,14_ = 9.83, p = 0.01).

## Discussion

We observed that the presence of endogeic earthworms near maize roots favoured the attraction of *H. megidis* entomopathogenic nematodes (EPNs) near wounded roots, both in a semi-field setting, and in an olfactometer experiment under climate-controlled conditions. Subsequent experiments showed that the earthworms’ facilitation effect was not mediated by earthworms directly enhancing plant defences, but most likely, through their restructuring of more favourable physicochemical soil conditions for EPN movement in the soil.

Cropping system significantly impacted the soil carbon–nitrogen ratio (CN) as well as plant traits related to biomass accumulation and reproduction. More nitrogen was present in polyculture soils (lower CN), suggesting that nodulated roots of bean plants actively fixed atmospheric nitrogen into the soil in a 14-week time. However, enhanced N-fixation did not promote maize growth. Instead, maize showed a general decrease in total biomass and produced grains that were almost a third lighter than those grown in simulated monoculture system. These unexpected results could indicate short-term negative interactions between this classic intercropping system composed of maize, squash and beans. As squash involved in this agroecosystem is more of a weed controller and water retainer^16^, it might be that competition by squash for water or nutrients prevailed over the beneficial transfer of N from bean to intercropped maize. Therefore, maize might have also simply taken more nitrogen from the soils as it grew larger in monoculture, therefore increasing the soil CN.

For both cropping systems, we also found that earthworms had no notable effect on maize performance. The 7-week long earthworm activity in the soil was probably insufficient for earthworms to significantly modify the chemical properties of the soil to have an impact on the growth of nearly adult maize plants. Indeed, the maize plants were approximately at stage V14–15 when the soil was inoculated with earthworms. Because the overall vegetative growth lasts 8–9 weeks (stages V1–V18), it is likely that earthworms were introduced too late in the mesocosms to significantly impact maize growth. Indeed, research on organic matter and nutrient dynamics in experimental fields indicates that earthworm effects on soil chemical properties may take of months to years^17^, explaining why no effect of earthworms were observed on short-term mesocosm soil. That said, contrary to the mesocosm experiments, the olfactometer-based experiments showed that maize plant growth traits were strongly enhanced by earthworm-worked substrate. Accordingly, we observed strong earthworm activity in the glass pots at the end of the bioassays (Supplementary Fig. S7). Endogeic earthworms are known for their positive influence on plant biomass from both laboratory and field experiments^18^, however, we here observed that their effect on plants seems to be context-dependent, with positive effects on short-term growth in an artificial subtract (i.e. compost and sand in the olfactometers), while having no effects on the medium term in our semi-field experiments.

Independently of earthworm effect on plant performance, EPNs were more efficient in reaching and infecting waxworm larvae near stressed roots, as well as being more attracted to the damaged plants, when soil contained earthworms. Taken together, the infectivity results of the mesocosm experiment and the olfactometer experiments suggest that induction by herbivores or JA promote the release of one or more nematode attractant(s) by maize roots, and that earthworms facilitate the chemical interaction between maize roots and EPNs. Three non-mutually exclusive hypotheses explaining the positive influence of earthworms on the response of EPNs to stressed roots may be: (1) a more favourable edaphic environment for EPNs to move through, (2) a higher diffusion rate of attractive volatile organic compounds in the soil due to earthworms burrowing^19^, and (3) a general enhancement of the volatile attractants that are released by the roots.

The laboratory experiments provided evidence to support the first two hypotheses that earthworm burrowing activity can enhance chemical communication between maize and EPNs as significantly more EPNs moved toward synthetic Eβc + CO_2_ emission in earthworm-worked soil compared to soil free of earthworm activity. It is known that Eβc diffusion and the subsequent response of EPNs can depend on soil texture and moisture^20^. EPN dispersal is also positively affected by soil moisture and increased structure^20^. Earthworms are likely to affect these soil properties through bioturbation and may thereby impact both Eβc diffusion as well as EPN recruitment in a positive way. To account for alternative ways earthworms might facilitate EPN movement, we also tested for the possibility that earthworms transported EPNs in their digestive tract. In support of this idea, it has been demonstrated that *Steinernema feltiae* EPNs can be transported by *Eisenia fetida* earthworms through phoresy, although such an activity decreased EPN infectivity potential by 70%^21^. To test how likely it is that phoresy contributed to EPNs movement, we measured how many EPNs were present in earthworm casts after being in close contact with each other (see Supplementary methods). We found that very few living EPNs were found in earthworm casts (Supplementary Table S2). In vivo transport of *H. megidis* by *A. icterica* therefore appears to be negligible.

Concerning the third hypothesis, EPN responses to earthworm presence were rather complex. While on one hand, in the first olfactometer experiment, we found that earthworms had no significant impact on Eβc emission, we also found that the earthworm mucus by itself enhanced Eβc emission, but it negatively impacted EPN attraction. While earthworms have been shown to have positive (or negative) effects on plant aboveground direct and indirect defence responses^8^, our findings that Eβc emission was not affected by earthworms, was contrary to our initial predictions, in which we expected that earthworms in close contact with the root system would readily activate plant defences (in this case Eβc emission). Nematodes appeared to not completely discriminate between plants with high or low Eβc emission. We propose two hypotheses to explain these observations. First, earthworm emission of CO_2_ may have contributed to blur or overtake the effect of specific root signals (or root-produced CO_2_), as EPN host-seeking behaviour is partly based on nonspecific cues such as CO_2_^22^. The possibility that the naturally-produced CO_2_ by *G. mellonella* larvae might have diffused faster than (synthetic) Eβc was also proposed in the study of Chiriboga et al.^20^, suggesting that CO_2_ could promote nematode host-seeking independently of the presence of other signals such as Eβc. An important role of invertebrate-produced CO_2_ alone seems rather unlikely in our study, because *H. megidis* were not more attracted to earthworm treatments than the earthworm-free herbivore-induced treatment.

It is also possible that EPNs might have been repelled by earthworm-emitted volatile compounds or other earthworm-derived exudates, as suggested by the mucus experiment. Endogeic earthworms can excrete large quantities of nitrogen in their mucus^23^, which may be one of the ways earthworms promote plant growth. While we did not see a significant effect of the mucus solution on root growth, we did see a positive effect of the mucus on Eβc production, but a repulsive effect on EPN recruitment. The mucus of earthworms is mainly composed of polysaccharides, amino-acids and water. Thus, increased Eβc in response to the N-rich might have mimicked the response of plants watered with N-rich nutrient solution, which have been shown to release more volatile organic compounds than unfertilized plants^15^. However, why EPNs moved away from mucus-watered plants by a factor of three as compared to controls remains puzzling. It is unlikely that nematodes were repulsed by the mucus itself, as earthworm contact with nematodes impacted positively EPN response in the bioturbation experiment. This leads to the conclusion that volatile compounds of earthworm mucus might have interfered with induced plant volatiles and disturbed EPN response. This idea needs to be tested in further studies.

In conclusion, we showed that the biological control potential of EPNs against root-feeding pests can be enhanced by the presence of earthworms in the soil. Earthworms generally also facilitate soil fertility and plant productivity^6^. While a recent review has shown that earthworms can reduce soil nematode abundance by 27%, such a negative effect of earthworms is generally cancelled out by the presence of plants in experiments^24^. Therefore, agroecosystems aimed at favouring local soil biodiversity and plant productivity while reducing pest load with a limited use of organic synthetic fertilizers and pesticides, would benefit from promoting endogeic earthworm presence. In addition, our studies with earthworms provide intriguing discrepancies between plant chemical defence traits and EPN behavioural responses, emphasizing the need for future studies on soil biogeochemistry and the biology of earthworms to include in the broad field of plant–insect interactions.

## Methods

### Mesocosm experiment

To study the effect of earthworms’ bioturbation activities on EPNs recruitment by maize roots, we performed an outdoor mesocosm experiment at the Botanical Garden of Neuchâtel, Switzerland. The mesocosms consisted of 50 × 50 × 30 cm wooden raised garden beds layered with a 1 mm diameter plastic mesh to prevent earthworm escape (Supplementary Fig. S1 in Supplementary material). Each mesocosm was filled with approximately 60 L of the A-layer of an Anthrosol (organo-mineral horizon) enriched with 10% compost and 10% sand. The initial soil was sieved once at 2 cm, and subsequently hand-sieved twice to remove all potential indigenous earthworms present. Before adding compost and sand, natural soil samples were collected, homogenized, dried at 40 °C for 48 h, sieved at 2 mm, and ground using agate mortars for subsequent physicochemical analyses. Specifically, we measured the particle size distribution (modified Robinson pipette method), the organic matter content through loss on ignition by weighing before and after burning 10 g of soil at 450 °C for 2 h, carbon to nitrogen ratio (CN) using an elemental analyser (FLASH2000, Thermo Fisher Scientific, Waltham, Massachusetts, United States); the pH in 1:2.5 soil to water ratio; the cation exchange capacity (CEC) following the cobaltihexamine chloride method; and the total phosphorous (using the Kjeldahl digestion method)^25^. The initial A-layer of the Anthrosol so was thus characterized as a silty-loamy soil (23% sand, 65% silt, 11% clay), with 7.06% organic matter content, 2.95% organic carbon, with a CN of 11.36, and with 19 ppm of total phosphorus content. The soil pH was 7.65 and the CEC was 5.0 cmolc/kg.

Four experimental treatments were tested (Supplementary Fig. S1): monocultures with and without the endogeic earthworm species *Allolobophora icterica*; and polycultures with and without the same earthworms. To reduce risks of interspecific variation in earthworms, we used a commercial strain of *A. icterica* supplied by the Ecotoxicology Department of National Institute for Agricultural Research (INRA Versailles, France).

The simulated monoculture consisted of three maize plants (*Zea mays* var. Delprim, UFA Delley Semences et Plantes, Delley-Portalban, Switzerland) per mesocosm, whereas one squash plant (*Curcubita pepo*, var. Rondini, Sativa Rheinau AG, Switzerland) and two bean plants (*Phaseolus vulgaris*, var. Neckargold, Sativa Rheinau AG) were growing with three maize plants in the simulated polycultures (Supplementary Fig. S1). In total we built 10 mesocosms per treatment (N = 40 mesocosms). All plants were sown early July and grown until mid-October 2018 before the onset of the experiment (Supplementary Fig. S2). Seven weeks after sowing, half of the mesocosms were inoculated with 15 earthworms each. The earthworms were standardized to 6 g of total fresh weight biomass per mesocosm.

In mid-October, the roots of one maize plant per mesocosm were mechanically damaged with a cork borer (punched three times near root area) and watered with 25 ml of a solution containing 500 μg (2.4 µmoles) of jasmonic acid (JA; ( ±)-Jasmonic acid, CAS Number: 77026-92-7, Sigma, St Louis, IL, USA) per plant to induce emission of volatile defence compounds^26^. JA is a growth phytohormone also called the “wound hormone” as it plays a central role in plant defence and has been shown to induce the release of Eβc in herbivore-attacked maize plants^22^. Mechanical damage was preferred over direct herbivory on roots to ensure damage reproducibility and to standardize the production of defence volatile compounds. Two days later, four *Galleria mellonella* (Lepidoptera: Pyralidae) larvae per mesocosm were placed in the soil as sentinel hosts to quantify EPN infection success. Specifically, in each mesocosm, the first two *G. mellonella* larvae were buried 5 cm deep in the soil and 5 cm away from the stem of a root-broken maize plant (damaged roots), while the second pair of larvae was placed in the same conditions, but close to the roots of an undamaged plant (control roots). Because late-instars *G. mellonella* larvae are immobile and highly susceptible to EPN infection, they have been extensively used for monitoring EPNs’ presence in soil^27,28^, as was done here. One day after *G. mellonella* addition, a solution of less than 2-week old 3000 infective juveniles *H. megidis* EPNs was inoculated at the centre of all mesocosms. The used *H. megidis* EPNs (Nematoda: Heterorhabditidae) were supplied by Andermatt Biocontrol AG, Switzerland, and reared on late-instar *G. mellonella* larvae in accordance with an in vivo rearing protocol described step by step^29^. Five days after EPN inoculation, all *G. mellonella* larvae were collected. Dead larvae were directly transferred into White traps to confirm infestation by EPNs, while living larvae were kept in soil-filled 5 × 6 × 4 cm plastic boxes for measuring potential EPN infection. Next, we collected plant traits related to biomass accumulation, including: total aboveground biomass, total vegetative height, and fitness, as the total biomass of all corncobs on each plant. Finally, a fraction of the soil was sampled in each mesocosm for fertility-related analyses; which included CEC and CN measures.

### Olfactometer-based bioassays

To dissect the interactive effect of root herbivory and earthworm presence near the roots of maize plants on EPN recruitment, a first (*four-arm*) olfactometer bioassay was conducted in controlled conditions of temperature, light and humidity (22 ± 2 °C day/16 ± 2 °C night, 55% RH, daytime 08:00 a.m.–06:00 p.m., 230 μmol/m^2^ s). The belowground olfactometer device (Fig. 2A), modified from Rasmann et al.^5^, consisted of a central glass chamber filled with white sand (Spielsand classic, Hamann Mercatus GmbH, Germany) extending in side arms connected to terminal glass pots (10 cm high, 15 cm diameter). Pots were filled with 1.2 L of soil (1/4 sand, and 3/4 standard potting soil; Ricoter, Aarberg, Switzerland, 10% relative humidity) as a standard for growing maize in the non-soil substrate when tested in olfactometers^30^. Three *A. icterica* earthworms were inoculated in two terminal pots (Fig. 2A). Simultaneously, one maize seedling was sown in each of the four terminal pots, and left to grow for 20 days (two-leaf to three-leaf stage). After 20 days of growth, which is considered as a minimum period for significant bioturbation of the soils^31^, three second instars of the banded cucumber beetle *Diabrotica balteata* (Coleoptera: Chrysomelidae) were added to two opposite glass pots containing the plants for the herbivore treatment setup (Fig. 3A). We used *D. balteata* instead of *D. v. virgifera* because of quarantine restrictions impeding the use of this species in the climate chambers in Switzerland. *D. balteata* is a generalist beetle that can feed on maize roots, and has been previously shown to induce maize plants to produce Eβc and attract EPNs^32^. Therefore, while slight differences might exist in terms of defence induction between the two *Diabrotica* species, they should be negligible and the results generalizable across Diabroticine beetles. Eggs of *D. balteata* were supplied by Syngenta Crop Protection (Stein, Switzerland) and larvae reared on a corn-based diet. Overall, the treatments followed a two-by-two factorial design experiment with the presence or absence of earthworms and root herbivores in each four-arm olfactometer (Fig. 2A). After three days, a solution of 2000 *H. megidis* infective juveniles was inoculated 5 mm below the sand surface in the middle of the central arena. After an additional 24 h, EPNs were retrieved from each side arm using the Baermann decantation funnel method. Next, roots were harvested, carefully washed and flash frozen in liquid nitrogen. Roots were ground in liquid nitrogen and Eβc production from each root system was measured using solid-phase microextraction (SPME) coupled to gas chromatography-mass spectrometry (GCMS) as described in Rasmann et al.^5^. Finally, for each plant, we scored root and shoot fresh biomass and plant height, measured as the longest leaf length from the ground. The same experiment consisting of 5 olfactometers each time was repeated four times for a total of N = 20 replicates.

A second (*two-arm*) olfactometer bioassay was performed in order to explore whether earthworm-emitted exudates via the epidermal mucus or faeces would directly interfere with the production of Eβc from maize roots, and the subsequent EPN movement. For this, maize seeds were sown in olfactometer glass pots (10 cm high, 5 cm diameter) with commercial substrate in similar conditions as described above (see Supplementary Fig. S3). Half of the plants were watered with tap water (control) while the other plants were watered with mucus solution. The mucus solution was obtained by caging 10 earthworms (adults and juveniles) in two 1 mm plastic mesh sieves in contact with each other’s open edge. The cage was submerged in 3 mm of tap water and left in darkness at room temperature (25 °C) for one hour, allowing earthworms to move into water and rub their skin against the sieve. The mucus of 20 earthworms (two cages) was collected to water 10 plants (equivalent of two earthworms per plant). The mucus solution (200 mL) was freshly prepared an hour before direct application onto the plants. All plants received the same volume of liquid at the same interval in order to keep substrate between plants as homogenously moist as possible. After 20 days, three second-instar *D. balteata* larvae were placed in every pot (controls and treatments) and left to feed on maize roots for three days. Connection with the olfactometer system was made 1 day before EPN inoculation, after which, a solution of 2000 infective juvenile EPNs was inoculated in the olfactometer central arena. After 24 h, EPNs were extracted from the two side arms of each olfactometer with the Baermann funnel method and counted under the microscope. Finally, root biomass and Eβc emissions were recorded as described above. The experiment was replicated 10 times.

A third (*two-arm*) olfactometer bioassay was performed to the test whether earthworm bioturbation activity in bulk soil affects nematode mobility alone, independently of earthworms being in contact with the maize root system. For this, soil-filled side arms and central arena and sand-filled terminal pots (10 cm high, 5 cm diameter) were assembled into a two-arm olfactometer (Fig. 4A). The same natural soil that was used for the mesocosm experiment at the Botanical Garden was used and sieved to 2 mm to ensure effective bioturbation and burrowing by earthworms. Ten soil samples were randomly taken from the soil stock, put on filter paper, emerged in water and left for decantation for 48 h to test the presence of any indigenous nematodes. None were observed and the soil was consequently not sterilized. Based on the study of Chiriboga et al.^20^ on diffusion of Eβc in different soil textures, soil and sand moisture were set respectively at 20% and 10% to maximise diffusion of volatiles. On the first day, three *A. icterica* earthworms were added to one half of olfactometer, and none in the other half. Earthworm bioturbation was restricted to half of the central arena by a 0.5 mm-mesh screen dividing it and by an anti-EPN mesh screen at the end of the side arms. Earthworms were left to work the soil for 4 days in climatic chamber (18 ± 2 °C, continuous darkness). Four days were considered enough time for three earthworms to properly burrow 0.5 L of soil. On day 5, the terminal pots were connected to olfactometer central system, and custom-made dispensers containing CO_2_ generating material (300 mg and sodium hydrogencarbonate and citric acid 3:1) and synthetic Eβc (300 μL, β-Caryophyllene, CAS Number 87-44-5, Sigma, St Louis, IL, USA) were prepared as described in Turlings et al.^22^, and inserted into the terminal pots filled with sand to ensure that EPN attraction was equally stimulated by both sides of the olfactometer (Fig. 4A). Five hours after inserting the dispensers, a suspension of 2000 *H. megidis* infective juveniles was inoculated in the central arena and left for 24 h, after which EPN presence in each arm was retrieved using Baermann funnels. The experiment was replicated eight times.

### Statistical analysis

All statistical analyses were performed on R^33^.

### Mesocosm outdoor experiment

We scored the probability of infection by dividing the number of larvae infected by EPNs around each plant by two. We then assessed the full interactive effect of culture type (two levels), earthworms (two levels), and root induction (two levels) on the probability of infection with generalized linear model analysis (GLM) with quasi-binomial distribution. We next performed the same GLM, but by comparing the interactive effect of earthworms and root induction, by splitting the data into monoculture and polyculture systems. Probabilities of infection scores were visualized using the library *popbio*^34^. The interactive effect of culture type and earthworms on CEC, CN, plant biomass and corn earcobs biomass was assessed using two-ways ANOVAs, followed by TukeyHSD post-hoc tests. For plant traits, we included mesocosm as a blocking effect in the model.

### Four-arm olfactometer bioassay

Analysis of variation in nematode recruitment across earthworms by root herbivory treatments was performed using generalized linear model analysis (GLM) with quasi-Poisson distribution to take data overdispersion into consideration, and by including the experiment date as a blocking factor in the model. Differences among treatments were assessed using analyses of deviance and F statistics. The analysis of the effect of herbivores, earthworms and their interactions on log + 1-transformed Eβc emissions were performed using two-way ANOVA, and by including experiment as blocking factor, and root biomass as covariate in the model. Differences among treatments were assessed using Tukey HSD tests. The effect of root herbivory and earthworms on plant biomass accumulation was assessed on the first principal component analysis (PCA) axis that included plant height, root and shoot fresh biomass (Supplementary Fig. S4).

### Mucus experiment

Analysis of variation in nematode recruitment across treatments was performed using GLM with quasi-Poisson distribution. Analysis of the effect of earthworm mucus on log + 1-transformed Eβc emissions and root biomass were performed using one-way ANOVAs.

### Bioturbation and synthetic Eβc olfactometer bioassay

The analysis of variation in nematode recruitment across earthworm presence/absence treatment was performed using GLM with quasi-Poisson distribution, and followed by analysis of deviance.

## Supplementary Information

Supplementary Information.

## Data Availability

Data underlying this article can be accessed on Dryad Digital Repository at 10.5061/dryad.ngf1vhhsk, and used under the Creative Commons Attribution licence.
