# Supplementary material for: Bioturbation by endogeic earthworms facilitates entomopathogenic nematode movement toward herbivore-damaged maize roots
Source: Sci Rep. 2020 Dec 4;10:21316. doi: 10.1038/s41598-020-78307-0 (PMC7718913; doi:10.1038/s41598-020-78307-0)
Supplement: Supplementary file 1 — Supplementary Information. [file 41598_2020_78307_MOESM1_ESM.docx]

Supplementary material for:

**Bioturbation by endogeic earthworms facilitates entomopathogenic nematode movement toward herbivore-damaged maize roots**

Sandrine Fattore^1^, Zhenggao Xiao^2^, Adrienne L. Godschalx^1^, Gregory Roeder^3^, Ted C. J. Turlings^3^, Renée-Claire Le Bayon^1^, Sergio Rasmann^1^

^1^Laboratory of Functional Ecology, University of Neuchâtel, Rue Emile-Argand 11, 2000 Neuchâtel, Switzerland

^2^ Institute of Environmental Processes and Pollution Control, School of Environmental and Civil Engineering, Jiangnan University, Wuxi 214122, China

^3^Fundamental and Applied Research in Chemical Ecology, Institute of Biology, University of Neuchâtel, Rue Emile Argand 11, 2000 Neuchâtel, Switzerland

**Supplementary methods**

*Phoresy experiment* - A phoresy experiment was carried out inspired by Campos-Herrera et al. (2006) to investigate the fate of EPNs after their passage through earthworm intestinal tract. Soil from the Botanical garden in Neuchâtel, Switzerland, was used as substrate, sieved to 2 mm, and exposed to 120°C for 60 minutes in an oven to kill any nematodes that might be naturally present in it. On day 1, intestinal content of 20 earthworms was emptied by placing them on filter paper in 15-cm diameter Petri dishes (10 individuals/dish) for 24 hours at 18±2°C in continuous darkness. On day 2, earthworms were rinsed and distributed in pairs in 20 60x15 mm Petri-dishes (filled each with 10g of soil (20% humidity). A solution of 2000 EPNs (IJ stage) was inoculated to each of the 10 Petri dishes (earthworm treatment). On the same day, the same nematode treatment was applied directly to 20 larvae of *G. mellonella* (one larva per dish) to test standard EPN infectivity without in vivo transport in earthworm intestinal tract (control). All Petri dishes were incubated at 22±2°C in continuous darkness for 24 hours. On day 3, earthworms were submerged and gently rinsed three times in tap water to remove any external nematode possibly present on the skin. Earthworms were individually transferred to new Petri dishes with damp filter paper to collect their first cast at 18±2°C in continuous darkness. An average of 24 to 36 hours was necessary to obtain a fully formed casts. Moth larvae were placed at the same time in new Petri dishes and maintained in continuous darkness at 22±2°C for 10 days to monitor infestation and register mortality. EPN-caused death was tested by placing all dead larvae on white traps. On day 4, all filter papers were collected, submerged in water, and left for 24 hours to extract nematodes out of earthworm casts. Earthworms were then individually transferred into soil-filled pots for 10 days to register potential post-nematode exposure mortality. On day 5, alive EPNs extracted from casts were counted under microscope. Prior to nematode counting, filter papers containing casts were removed from funnel, left for 10 minutes on the inclined surface of the funnel to drain the excess water before being transferred to new Petri dishes. One new *G. mellonella* larvae was placed per dish. When detected during counting, every survivor nematode was individually pipetted from extraction water and inoculated back to its filter paper to test *H. megidis* efficacy (motility and infectivity) on new larvae. The Petri dishes were incubated at 22±2°C in darkness for another 10 days to monitor larvae mortality. On day 15, living larvae were considered as non-infected while dead ones were placed on white traps to determine whether death was caused by EPN. The number of EPN that survived gut passage as well as success or failure of last stage infestations were recorded.

**Table S1** Two-way ANOVA table for testing the effect of culture (polyculture versus monoculture) and earthworm (presence or absence) treatments on total plant biomass and total biomass of corn ears (fruits).

| Variable | Treatment | Df | SumSq | F value | p value |
| --- | --- | --- | --- | --- | --- |
| Plant biomass | Earthworm (EW) | 1 | 40 | 0.19 | 0.66 |
|  | Culture type (C) | 1 | 3608 | 16.81 | **<0.001** |
|  | Mesocosm | 18 | 12459 | 3.23 | **<0.001** |
|  | EW * C | 1 | 590 | 2.75 | 0.10 |
|  | Residuals | 96 | 20598 |  |  |
| Corn ears biomass | Earthworm (EW) | 1 | 20 | 0.38 | 0.54 |
|  | Culture type (C) | 1 | 624 | 11.78 | **0.001** |
|  | Mesocosm | 18 | 1502 | 1.58 | 0.08 |
|  | EW * C | 1 | 2 | 0.03 | 0.86 |
|  | Residuals | 96 | 5087 |  |  |

**Table S2** Raw data of phoresy experiment. **a**. Result table of earthworm treatment (passage in *Allolobophora icterica* intestinal tract). **b**. Result table of control (no earthworm gut passage/direct test on insect larva *Galleria mellonella*).


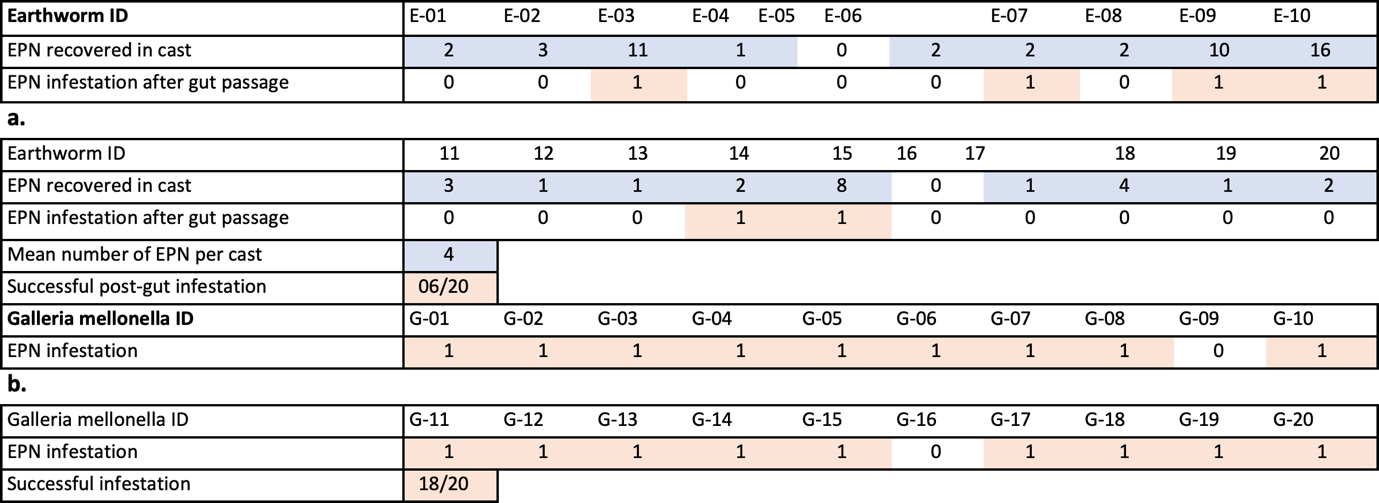


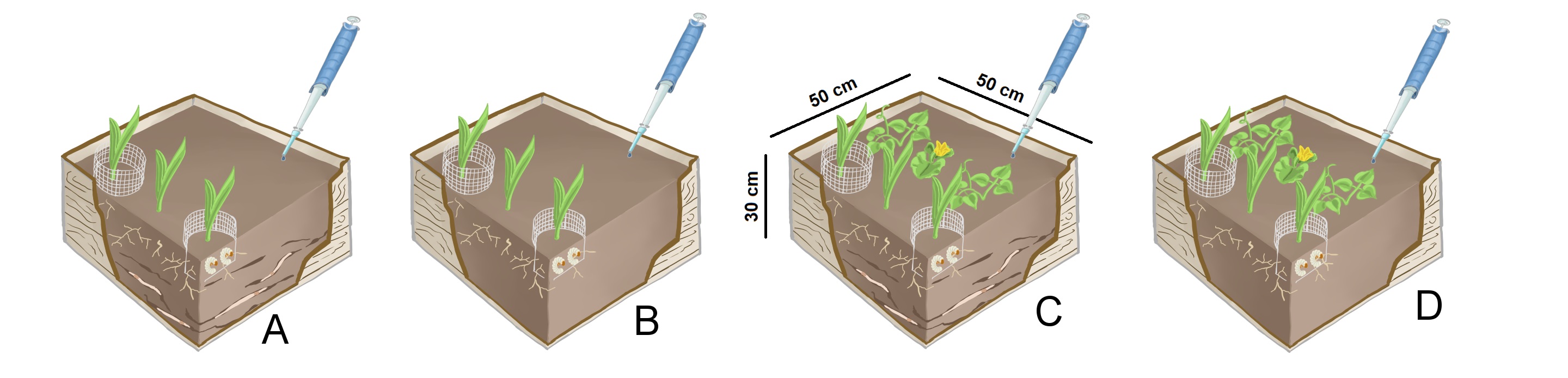


**Figure S1** Experimental design of the outdoor mesocosm experiment. Four treatments were included: A) Monoculture with earthworms, B) monoculture without earthworms, C) polyculture with earthworms, and D) polyculture without earthworms. Earthworms consisted of placing 20 *Allolobophora icterica* endogeic earthworm individuals in each treatment boxes. Simulated monoculture consisted of three maize plants (*Zea mays* var. Delprim) per box, whereas only one squash plant (*Curcubita pepo* var. Rondini) and two bean plants (*Vicia faba* var. Neckargold) were growing with three maize plants in the simulated polyculture.


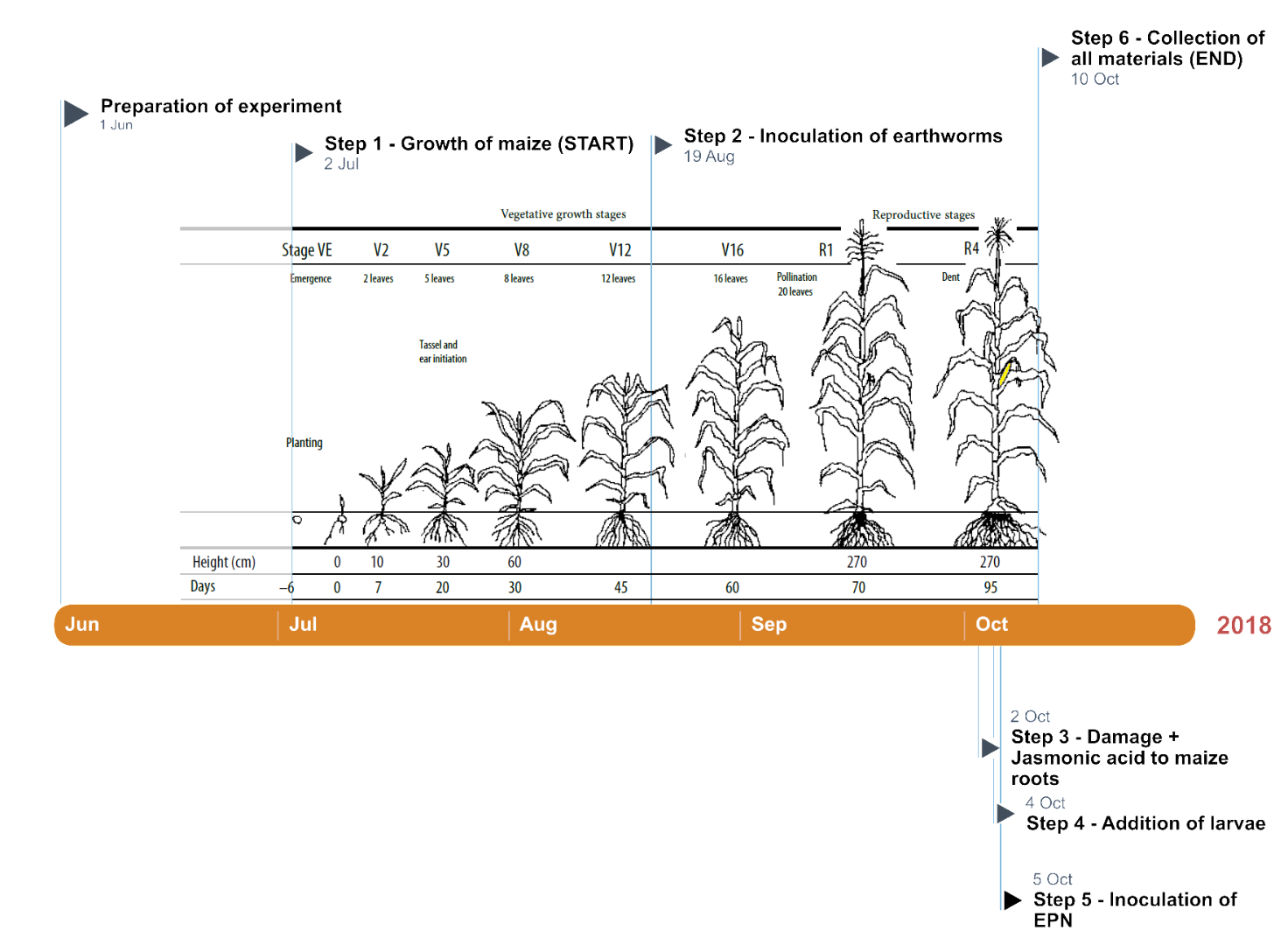


**Figure S2** Timeline of semi-field experiment. Modified from O'Keeffe (2009).


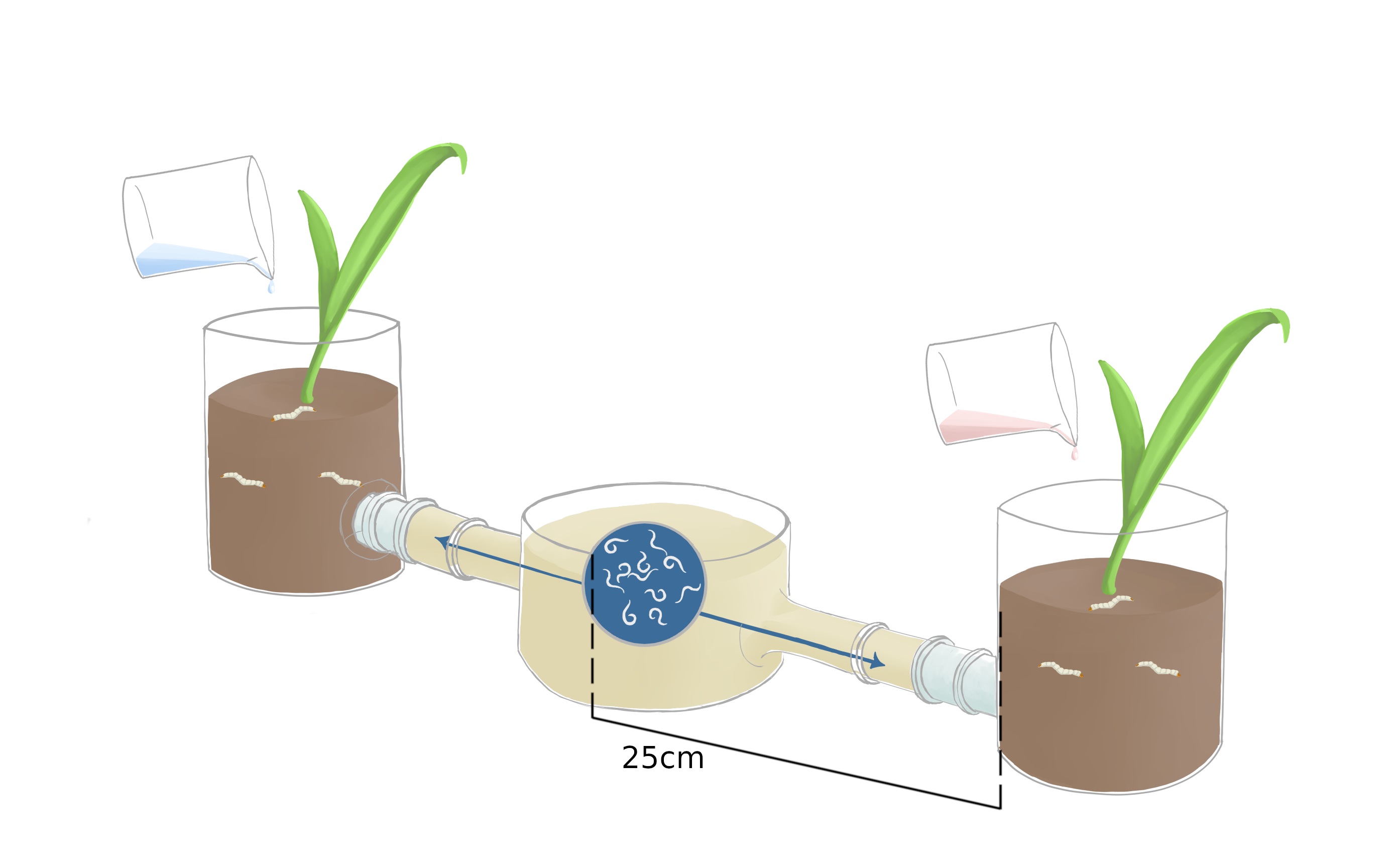


**Figure S3** Experimental design of mucus olfactometer bioassay. One maize plant per pot, attacked by larvae of *Diabrotica balteata*. **A.** Maize watered with tap water (control) **B.** Maize watered with mucus solution (mucus treatment).


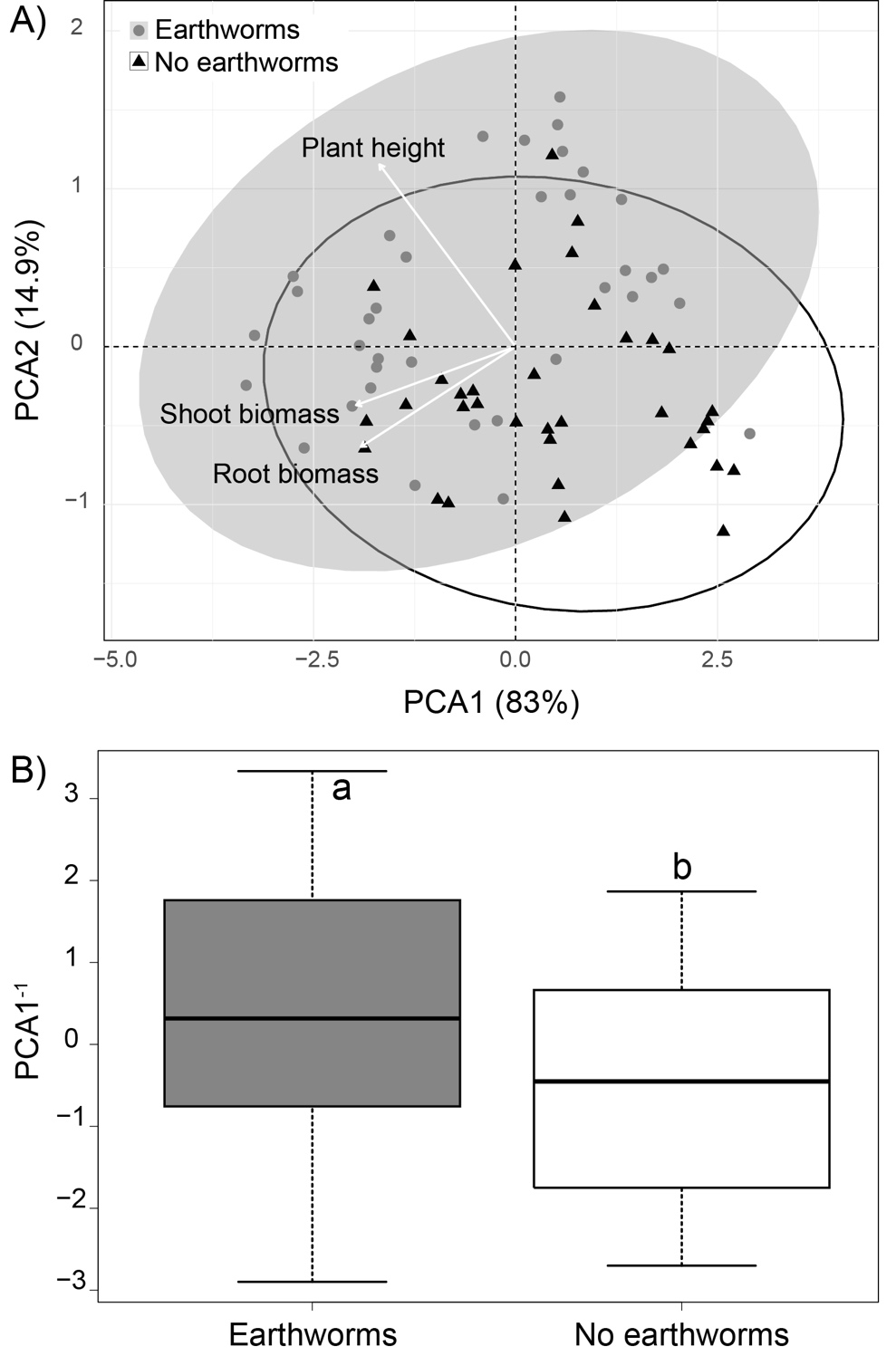


**Figure S4 Effect of earthworms on plant growth**. Shown is A) a principal component analysis (PCA) ordination of maize plants height, root and shoot fresh weight, when in the presence (grey ellipse, and grey dots), or in the absence (open ellipse, and black triangles) of *A. icterica* earthworms. B) Boxplot representation of the average effect of earthworms’ presence on the inverse first PCA axis. Positive values in this case, indicate a positive effect of earthworms on maize biomass accumulation. Letters above boxplots indicate significant difference after linear model (p < 0.05).


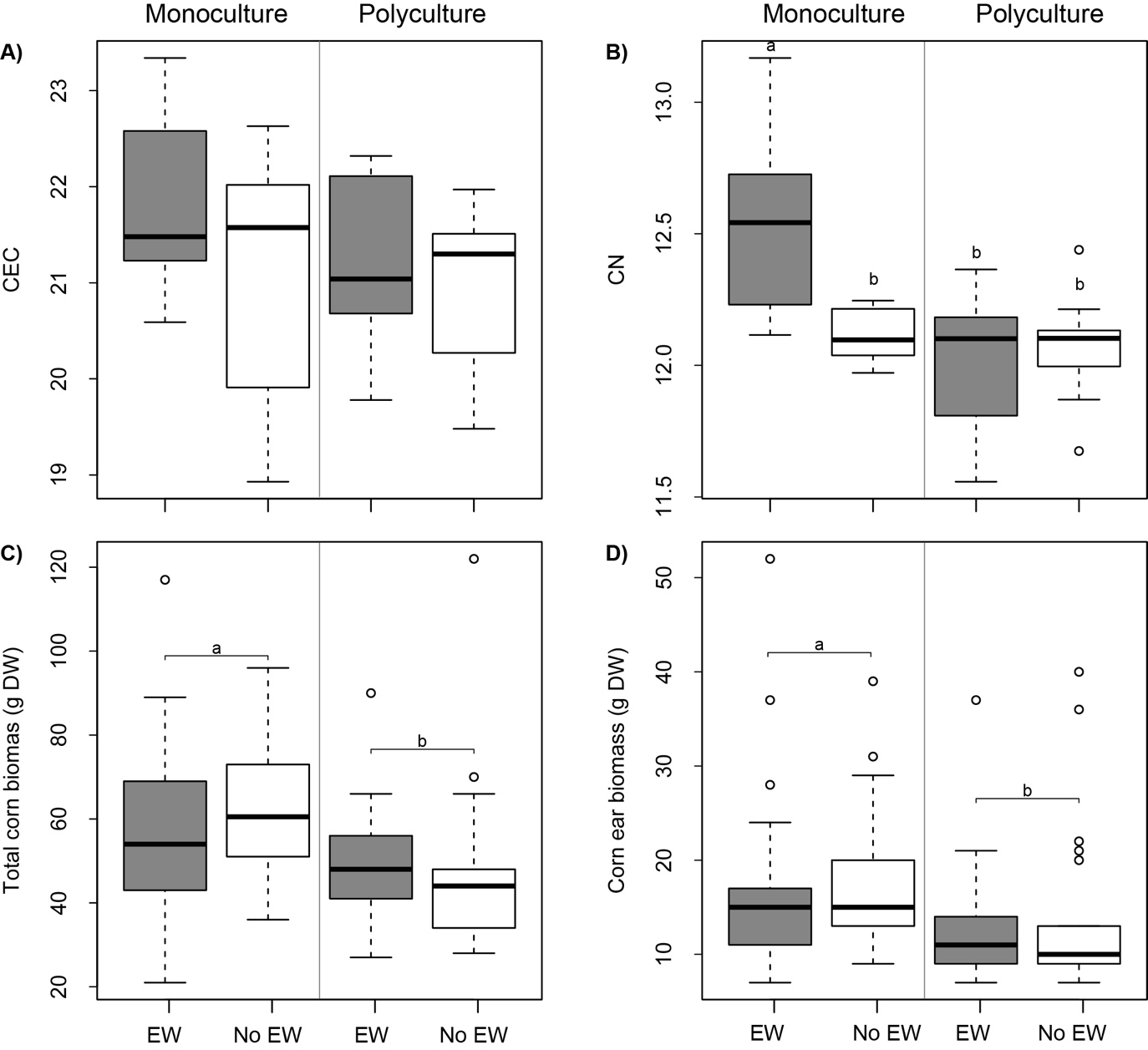


**Figure S5.** Edaphic component and plant traits under different cultural and earthworm treatments. Boxplots show average values for A) cation exchange capacity (CEC), B) total organic carbon to total nitrogen ratio (CN) of soils that came from either monoculture of only maize plants, or polyculture of maize, squash and beans, that were crossed with the presence of *A. icterica* earthworms (grey boxes) or their absence (open boxes). Different letters above boxes indicate significant differences among treatments (TukeyHSD, p < 0.05).

**Figure S6** Effect of earthworm mucus on belowground tritrophic interactions. Boxplots show A) average entomopathogenic nematode (EPN) attraction toward maize roots infested by *Diabrotica* *balteata* larvae, and when roots were watered with tap water (Control, open boxes), or with a mucus solution of *A. icterica* earthworms (Mucus, grey boxes). B) Average (*E*)-β-caryophyllene production, and C) root biomass from the same plants. Asterisks mean significant differences between treatments (p < 0.05).


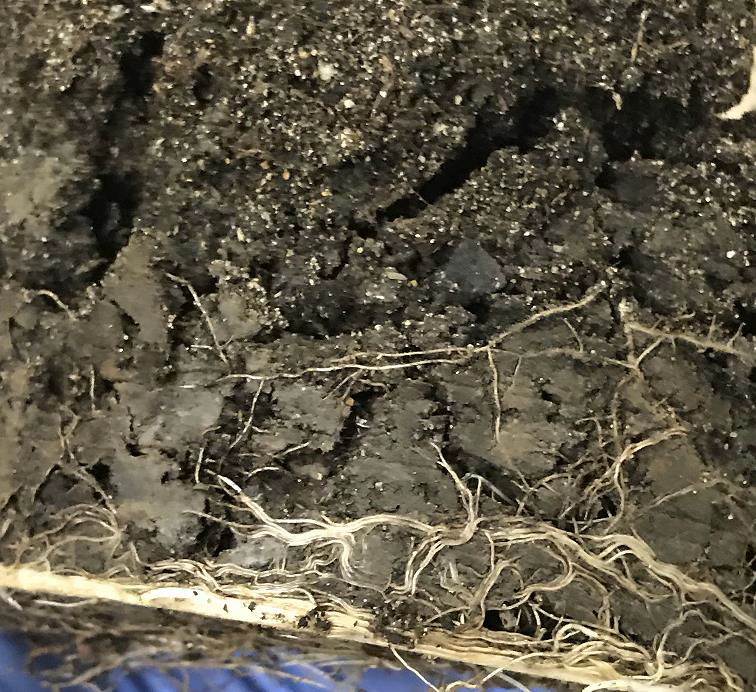


A)


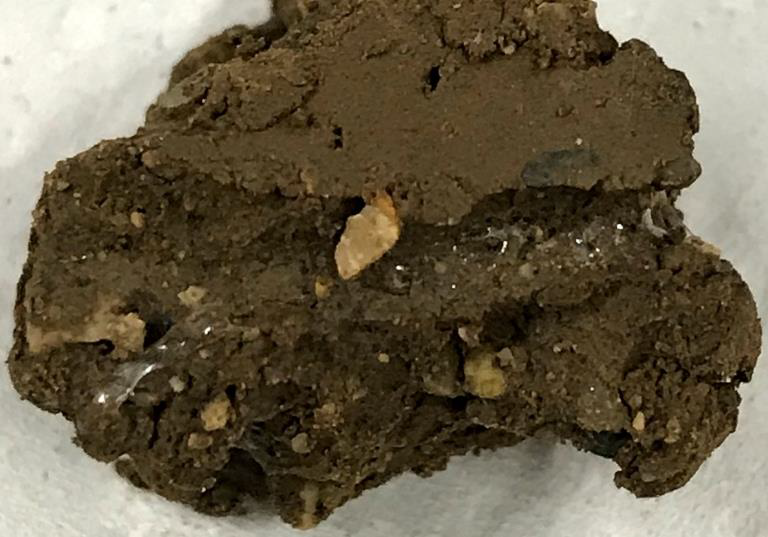


B)

**Figure S7** Earthworm-worked soil in mesocosms experiments. A) Extensive horizontal and vertical burrowing by *A. icterica* in olfactometer pots after 28 days. Photograph by Adrienne Godschalx. B) Galleries of *A. icterica* earthworm lined with mucus. Photograph by Mario Rodriguez.
